# The mitochondrial‐derived peptide MOTS‐c is a regulator of plasma metabolites and enhances insulin sensitivity

**DOI:** 10.14814/phy2.14171

**Published:** 2019-07-10

**Authors:** Su‐Jeong Kim, Brendan Miller, Hemal H. Mehta, Jialin Xiao, Junxiang Wan, Thalida E. Arpawong, Kelvin Yen, Pinchas Cohen

**Affiliations:** ^1^ Leonard Davis School of Gerontology University of Southern California Los Angeles CA

**Keywords:** High‐fat diet, insulin resistance, metabolomics, MOTS‐c

## Abstract

MOTS‐c is an exercise mimetic and improves insulin sensitivity in aged and diet‐induced obese mice. Although plasma markers are good markers for the metabolic condition, whether MOTS‐c changes plasma markers in diet‐induced obese mice has not been examined. Here, we used an unbiased metabolomics approach to examine the effect of MOTS‐c on plasma markers of metabolic dysfunction. We found that three pathways – sphingolipid metabolism, monoacylglycerol metabolism, and dicarboxylate metabolism – were reduced in MOTS‐c–injected mice. Interestingly, these pathways are upregulated in obese and T2D models. MOTS‐c improves insulin sensitivity and increases beta‐oxidation to prevent fat accumulation in DIO mice through these pathways. These results provide us a better understanding of the mechanism of how MOTS‐c improves insulin sensitivity and reduces the body weight and fatty liver and opens a new venue for further study.

## Introduction

MOTS‐c is a 16‐amino acid peptide, which is encoded in the 12S rRNA region of the mitochondria (Lee et al., 2015). It is measured in plasma and multiple tissues including muscle, brain, and liver (Lee et al., 2015). MOTS‐c levels are correlated with insulin resistance in lean, not obese, individuals and circulating MOTS‐c levels are reduced in obese male children and adolescents, but not in obese females (Cataldo et al., 2018; Du et al., 2018). MOTS‐c levels are correlated with markers of insulin resistance and obesity including BMI, waist circumference, waist‐to‐hip ratio, fasting insulin level, HOMA‐IR, HbA1c (Du et al., 2018). In addition, MOTS‐c levels are correlated with endothelial function in humans (Qin et al., 2018). The effects of MOTS‐c include increased glucose utilization and fatty acid oxidation, decreased oxidative phosphorylation, increased endogenous AICAR levels, and AMPK activation (Lee et al., 2015). MOTS‐c suppresses ovariectomy‐induced osteoporosis via AMPK activation (Ming et al., 2016). MOTS‐c improves osteoporosis via the TGF‐β/Smad pathway (Hu and Chen, 2018). MOTS‐c administration improves the insulin sensitivity of old mice by increasing glucose uptake in the muscle (Lee et al., 2015). MOTS‐c administration in high‐fat diet‐fed mice decreases the weight gain by increasing energy expenditure and significantly decreases the fat accumulation in the liver (Lee et al., 2015). The levels of insulin also were lower in MOTS‐c–injected mice, suggesting that MOTS‐c improves the insulin sensitivity in high‐fat diet‐induced mice (Lee et al., 2015).

Metabolomics studies in plasma samples from subjects with type 2 diabetes identified significant changes in various metabolite classes. Elevated plasma deoxysphingolipids and ceramide are risk predictors of type 2 diabetes (Kirwan, 2013; Mwinyi et al., 2017). Glycine and lysophosphatidylcholine, and Glyoxylate levels are predictors for impaired glucose tolerance and type 2 diabetes (Wang‐Sattler et al., 2012; Nikiforova et al., 2014). The complete plasma metabolome could provide insight to the mechanisms of insulin‐sensitizing molecules. Given the vast roles in energy utilization, sphingolipid, monoacylglyercol, and dicarboxylic acid metabolism could be potently modulated by insulin‐sensitizing molecules.

Altered sphingolipid metabolism and more specifically enhanced ceramide and sphingosine 1‐phosphate (S1P) levels in plasma lead to progress of type 2 diabetes and insulin resistance, contributing to metabolic syndrome (Kirwan, 2013; Fayyaz et al., 2014; Raichur et al., 2014; Turpin et al., 2014). High‐fat diet selectively upregulates expression of CerS6, the ceramide synthetic enzyme, and enhances ceramide production (Turpin et al., 2014). Ceramide induces insulin resistance by antagonizing insulin‐mediated PI3K/AKT signaling, which attenuates glucose metabolism (Turpin et al., 2014). Ceramide directly inhibits mitochondrial electron transport and indirectly suppresses beta‐oxidation (Turpin et al., 2014). Inhibition of beta‐oxidation reduces fatty acid disposal, promoting accumulation of excessive triglyceride in lipid droplets (Turpin et al., 2014). Increased levels of glycosphingolipids are associated with resistance to insulin signaling as manifested by reduced insulin‐stimulated glucose uptake (Zhao et al., 2007). Reduction of glycosphingolipid levels by inhibitors of synthesis, treatment with degrading enzymes, or knockout of biosynthetic enzymes in mouse models diminished insulin resistance, suggesting a crucial role for this type of sphingolipids (Zhao et al., 2007). Recent studies showed sphingosine 1‐phosphate (S1P) plays important roles in insulin resistance (Hla and Dannenberg, 2012; Maceyka et al., 2012; Fayyaz et al., 2014). Elevation of S1P plasma levels is recognized as a critical characteristic of both human and rodent obesity (Kowalski et al., 2013). Plasma S1P levels are elevated in two animal models of type 1 diabetes (Fox et al., 2011). An S1P receptor antagonist prevents the onset of diabetes in streptozotocin diabetes mouse models and S1P receptor knockout mice showed lower blood glucose levels and reduced beta‐cell death (Imasawa et al., 2010). S1P levels correlate with insulin resistance (Fox et al., 2011). S1P binds to the S1P_2_ receptor resulting in an inactivation of insulin‐mediated PI3/AKT signaling leading to a decrease of glucokinase expression and GSK‐3 beta activation (Osawa et al., 2005). This results in a diminished glycogen synthesis in the liver (Babenko and Kharchenko, 2015). S1P binds to a S1P_3_ receptor, leading to the formation of IL‐6, which mediates insulin resistance in the muscle (Ross et al., 2013). On the other hands, S1P increases beta‐cell survival via AMPK and AKT activation (Laychock et al., 2006; Mastrandrea et al., 2010). S1P has divergent roles in insulin resistance.

Monoacylglycerols are generated from diacylglycerols by lipase. The activity of membrane‐bound lipoprotein lipases at the tissues (eg. adipose, muscle, and liver) is modulated by angiopoietin‐like proteins (ANGPTLs) (Zhang, 2016). Recent data indicate that ANGPTL3, 4, and 8 serve as a potent inhibitor of the LPL enzyme (Zhang, 2016). ANGPTL3 and 4 have many common features (Lee et al., 2009). ANGPTL4 suppresses the LPLs activity and subsequently inhibits the uptake of the fatty acids and monoacylglycerol by underlying tissues, including adipose tissue, skeletal and cardiac muscle (Lee et al., 2009). ANGPTL4 plays a relevant role in type 2 diabetes mellitus and in the metabolic syndrome (Gusarova et al., 2018). In mice, ANGPTL4 decreases blood glucose and improves glucose tolerance (Xu et al., 2005). Systemic administration of ANGPTL4 suppresses food intake and body weight gain via suppression of hypothalamic AMPK activities (Kim et al., 2010). ANGPTL8 is considered a novel but atypical ANGPTL family member, because of its structure (Quagliarini et al., 2012). ANGPTL8 is a hepatocyte‐derived circulating factor that regulates plasma triglycerides levels and a mediator of trafficking of fatty acids to adipose tissue (Quagliarini et al., 2012).

Dicarboxylic acids (DCAs) are formed from omega oxidation of monocarboxylic acids, mainly in the liver and kidney (Wanders et al., 2011). The beta‐oxidation of DCAs is independent of normal beta‐oxidation but still occurs in both mitochondria and peroxisome (Wanders et al., 2011). DCAs are suitable energy substrate with characteristics intermediate between glucose and fatty acids (Wanders et al., 2011). When glucose metabolism is impaired, DCAs can be a fuel substrate immediately available for tissue energy requirements (Mingrone et al., 2013). Increases in dicarboxylates are generally thought to indicate dysfunction of normal mitochondrial and peroxisomal beta‐oxidation (Yamakaya, 1950; Bergstrom et al., 1954). Different genetic diseases in humans in which either peroxisomal or mitochondrial fatty acid oxidation are impaired, omega oxidation induced as a rescue mechanism to allow the breakdown of fatty acids (Wanders et al., 2011). Therefore, DCAs were implied that it can be a useful alternative substrate in parenteral nutrition, sparing glucose utilization and increasing glycogen stores in those clinical conditions like type 2 diabetes, where reduced insulin‐induced glucose uptake and oxidation are observed. Indeed, studies in animals and humans with type 2 diabetes showed that oral administration of DCAs (sebacic acid) improved glycemic control, probably by enhancing insulin sensitivity, and reduced hepatic gluconeogenesis and glucose output (Iaconelli et al., 2010). Moreover, DCAs (dodecanedioic acid) intake reduced muscle fatigue during exercise in subjects with type 2 diabetes, suggesting an improvement of energy utilization and metabolic flexibility (Salinari et al., 2006). Metabolically healthy skeletal muscle possesses the ability to switch easily between glucose and fat oxidation in response to homeostatic signals (Kelley, 2005). In type 2 diabetes and obesity, skeletal muscle shows a great reduction in this metabolic flexibility (Kelley, 2005).

Although MOTS‐c showed significant effects on diet‐induced obesity and insulin sensitivity, the precise mechanisms are still being investigated. MOTS‐c has been shown to increase glucose uptake in muscle in response to insulin, and MOTS‐c may play other roles in modulating metabolites or signaling pathways to improve insulin sensitivity in high‐fat diet‐fed mice (Lee et al., 2015). Here, we investigate the hypothesis that MOTS‐c alters plasma metabolites, which are associated with insulin resistance, to improve insulin sensitivity. To address this question, we performed metabolomics of the plasma from mice injected with MOTS‐c.

## Materials and Methods

### Ethical approval

All animal procedures were carried out according to the University of Southern California’s Animal Care and Use Committee and Animal Research.

### Animals and diets

C57/6NJ male mice (17 weeks old) were obtained from Jackson Laboratories and mice were fed a high‐fat diet since they were 5 weeks old with continuous access to water. High‐fat diet nutrient composition consisted of 3.08 kcal/g, 60% fat, 15% protein, and 25% carbohydrates. Animals were fed the same high‐fat diet throughout the 3‐day treatment duration. A total of 14 mice (*n* = 7 per group) were injected for this study, and a total of 12 mice (*n* = 6 per group) were analyzed for metabolomics. Body weight was measured daily in the morning, while blood glucose was measured at the start and finish of 3‐day treatment. Blood glucose samples were collected via the small incision of the tail vein and quantified using FREESTYLE LITE BLOOD GLUCOSE METER.

### Mitochondrial‐derived peptide administration

After receiving the mice, animals were housed for 6 days under staff supervision. On the sixth day, mice were intraperitoneally injected with 2.5 mg/kg MOTS‐c (Genscript) or water twice a day for 3 consecutive days. MOTS‐c was freshly prepared in Milli‐Q Water immediately prior to administration. On the day after final peptide treatment, mice were euthanized by isoflurane and samples were immediately collected and frozen at −80°C for subsequent experiments.

### Metabolomics

Metabolomics of mice plasma was performed by Metabolon (Durham, NC). In general, mice plasma samples (100 *μ*L) were subjected to protein precipitation and separated into four extracts for four ultra‐high‐performance liquid chromatography/tandem accurate mass spectrometry methods: (1) ultra‐high‐performance liquid chromatography‐tandem mass spectrometry (UPLC‐MS/MS; positive ionization), UPLC‐MS/MS (negative ionization), UPLC‐MS/MS polar platform (negative ionization), and gas chromatography‐mass spectrometry (GC–MS). Generated data are then compared to Metabolon’s reference library, which is made up of known chemical standard entries based on retention time, molecular weight, preferred adducts, in‐source fragments, and MS spectra.

### Meso scale discovery

Mouse plasma was collected from the mice treated with water and MOTS‐c. Insulin and leptin levels in the plasma were determined using the MSD custom 8‐plex kit (cat. # N051A, MESO SCALE DISCOVERY, Rockville, MD) according to the manufacturer’s protocols.

### RNA extraction and RT‐qPCR

Total RNA was extracted from cells and tissues using Direct‐zol RNA MiniPrep Plus (ZYMO RESEARCH, Irvine, CA). One milligram of RNA was used for reverse transcription by using SuperScript IV Reverse Transcriptase and oligo dT_20_ primers (ThermoFisher Scientific) according to the manufacturer’s instruction. Ssoadvanced Universal SYBR green supermix was used to amplify cDNA and quantify the relative gene expression analysis. The 2^−ΔΔCT^ method is used for relative gene expression analysis. This method shows the fold increase (or decrease) of the target gene in the test sample relative to the calibrator sample and is normalized to the expression of a reference gene.

### Statistics

To identify the pathways that MOTS‐c modulates, relative metabolite abundances were compared between the MOTS‐c–treated mice and water‐treated mice using multivariate analyses by Metabolon. Data were subjected to hierarchical clustering analysis and principal component analysis (PCA), whereby data were transformed via orthogonal linear transformation into principal components to visualize distinct clusters between the groups. Differential metabolite levels were quantified by relative abundance and median scaled to one. Distinction in relative abundance was evaluated by matched pair *t*‐tests, with a false discovery threshold of *q* < 0.10 to correct for multiple comparisons present in metabolomics studies. Fold difference was determined by dividing the relative metabolite abundance in the MOTS‐c group by the relative abundance in the water group. Outputs can be interpreted as fold change in MOTS‐c treatment compared to water treatment. Scores less than one indicate significantly lower MOTS‐c metabolite differences compared to the control group, whereas scores greater than one suggest significantly higher MOTS‐c metabolite differences. Data were considered significant at *P* < 0.05 with *q* values below *q* < 0.1.

We also performed our own statistical analysis in three stages (Fig. 2 and Fig. S1). First, we conducted PCA and hierarchical clustering analysis for the complete set of 550 metabolites measured. Second, to further reduce the dimensions of the data and provide for greater interpretability, we implemented a biologically informed approach and conducted pathway analysis by Metabolon. This enabled us to select 52 metabolites that have shown functional relevance to the hypothesized pathways involved. Third, we conducted PCA and hierarchical clustering on the subset of 52 metabolites identified through pathway analysis.

PCA is a statistical approach with which to reduce the dimensions of the dataset while preserving critical variation in the data. For PCA, we used R v3.5.0 and the Factoextra package. PCA was conducted after normalizing the data in order to ensure equal contribution of each metabolite to the analysis. Then, we extracted the eigenvalues to construct scree plots and to calculate the amount of variation represented by each PC. Using all 550 metabolites, the first three PCs explained 27.3%, 18.8%, and 14.6, respectively, or 60.6% cumulatively (Fig. S1). Next, we assessed PCA individual and variable plots. The individual PCA plot shows the distribution of each treatment and control mice when plotting the first two PCs. The variable PC plot shows the relationships between all variables, whereby those that are positively correlated are grouped together. In contrast, variables that are negatively correlated are positioned on opposite sides of the plot centroid. The distance between the variables and the centroid indicates the quality of the variables on the factor map. Initially, the variable plot was uninterpretable due to the sheer quantity of variables used in PCA. Hence, to use an empirically informed approach to reduce the number of variables, we used the top three pathways identified by Metabolon’s statistical analysis. Paths identified enables us to isolate 52 metabolites that were found to have biological significance. Using the subset of 52 metabolites, we conducted a subset PCA. The first three PCs explained 41.1%, 21.5%, and 13.3%, respectively, or 75.9% cumulatively (Fig. 2). We assessed individual and variable PCA plots.

For hierarchical clustering analysis, we used R v3.5.0 and the gplots package. We produced heatmaps and dendograms, for the complete metabolite set (Fig. S1).

For blood glucose, body weight, and food intake analyses, data were analyzed using GraphPad Prism (San Diego, CA, USA). Student *t*‐test examined mean differences between the MOTS‐c group and water group. Data are represented as the mean ± SEM and *P* < 0.05 was considered significant.

## Results

### Metabolic characterization of MOTS‐c–treated mice

**Figure 1:**
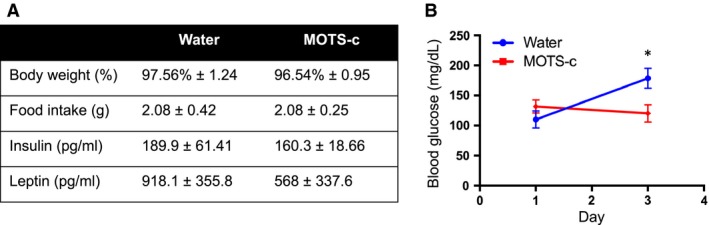
Characteristics of MOTS‐c injected diet‐induced obese (DIO) mice. C57BL/6J mice (17‐weeks old) were fed a high‐fat diet starting at 5 weeks of age. MOTS‐c peptide injections (2.5 mg/kg; IP; BID) were performed for 3 days. (A) Body weight, food intake, insulin, leptin, and (B) Blood glucose levels were measured. Data are reported as mean ± SEM of six mice per group. ^*^ *P* < 0.05.

We analyzed blood glucose and body weight differences between diet‐induced obese mice (DIO) administered with MOTS‐c or water for 3 days. There were no differences in body weight change between MOTS‐c and water treated, and mice did not differ in food intake throughout the 3‐day treatment duration (Fig. 1A). Insulin and leptin levels trended lower in the MOTS‐c treated group. There was, however, a significant difference in blood glucose (mg/dL) of mice between MOTS‐c and water treatments after 3 days (Fig. 1B). Taken together, it suggests MOTS‐c increases insulin sensitivity in high‐fat diet‐induced obese mice.

### Distinct metabolic profiles in MOTS‐c–treated mice

**Figure 2:**
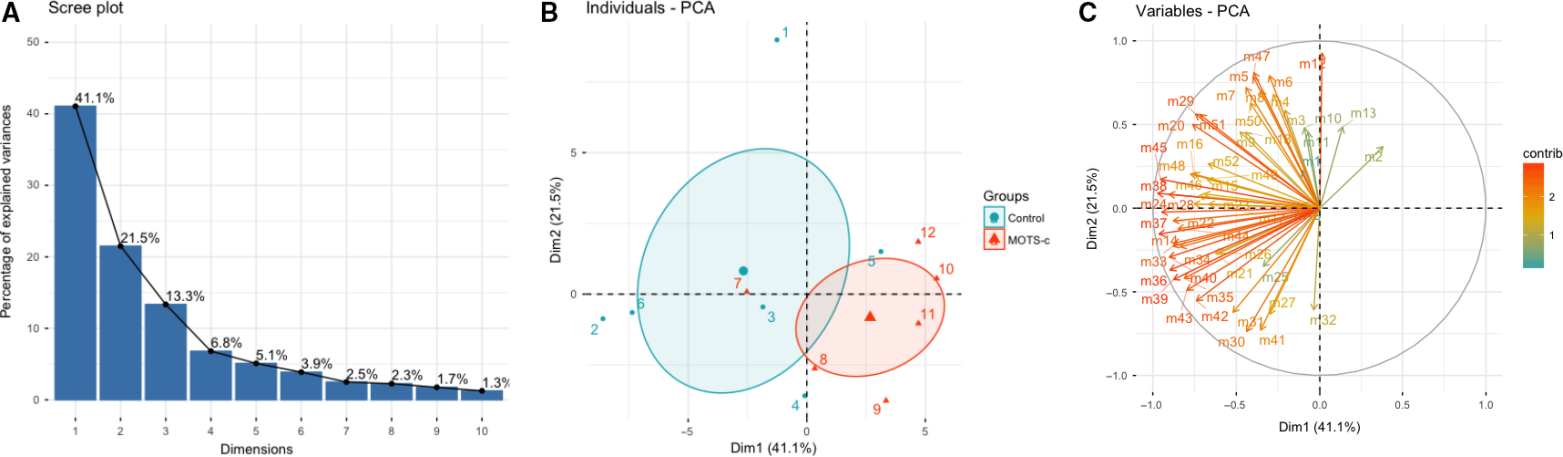
The selected plasma metabolites differences between water and MOTS‐c injected DIO mice were distinct. (A) Variance percentage scree plots (B) 2D individuals factor map (C) 2D variables factor map.

Distinct metabolic profiles were clear between MOTS‐c and water‐treated mice. Principal component analysis (PCA) of MOTS‐c and control samples revealed discrete clustering in relative metabolite abundance (Fig. 2A–C and Fig. S1A–C). Furthermore, hierarchical clustering analysis (HCA) shows MOTS‐c and water treatments partially segregate into clusters (Fig. S1D). From the results of PCA and HCA, MOTS‐c treatment induced a significant shift in metabolites and represents a systematic change in pathway utilization. Three pathways related to insulin sensitivity and lipid metabolism were altered by MOTS‐c treatment: (1) sphingolipid metabolism, (2) monoacylglycerol metabolism, and (3) dicarboxylate metabolism. Subsequent analyses were performed to elucidate the extent to which MOTS‐c modulated specific pathways involved in metabolism, in particular, insulin sensitivity to understand the roles of MOTS‐c in DIO mice.

### Sphingolipid metabolism

**Figure 3:**
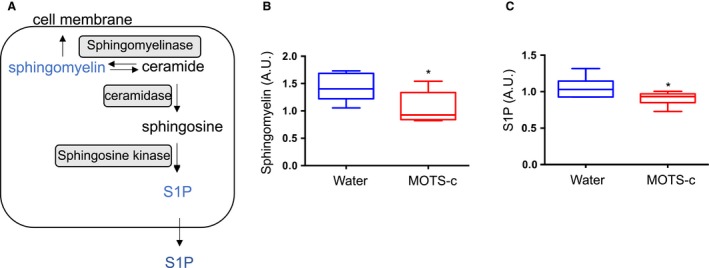
MOTS‐c administration decreases S1P levels in the plasma. (A) Schematic diagram of sphingolipid metabolism (B) Sphingomyelin and (C) sphingosine 1‐phosphate (S1P) levels in plasma from DIO mice injected with either water or MOTS‐c (*N* = 6/group) Data are reported as mean ± SEM of six mice per group. ^*^ *P* < 0.05.

Sphingolipid metabolism generates metabolites related to type 2 diabetes and insulin action (Meikle and Summers, 2017). Ceramide is a byproduct of sphingolipid metabolism (Fig. S2A) and is involved in several cascades that promote cell death and inflammation, mechanisms by which insulin resistance emerges (Turpin et al., 2014). Sphingosine 1‐phosphate (S1P) is generated by the ceramide‐sphingosine‐S1P pathway (Fig. 3A). Elevated ceramide and S1P levels were observed in T2D and obese subjects (Kirwan, 2013; Fayyaz et al., 2014; Turpin et al., 2014; Raichur et al., 2014). S1P inhibits insulin‐mediated AKT signaling in the liver and muscle via the S1P receptor (Osawa et al., 2005). MOTS‐c pointedly altered sphingolipid metabolism in a way to suggest protection against insulin resistance. In particular, several sphingolipid metabolites were decreased in mice given MOTS‐c (Table 1), such as sphinganine‐1‐phosphate (*P* = 0.021; fold change = 0.73), palmitoyl sphingomyelin (d18:1/16:0) (*P* = 0.0067; fold change = 0.77), stearoyl sphingomyelin (d18:1/18:0) (*P* = 0.0043; fold change = 0.63), sphingomyelin (d18:1/18:1, d18:2/18:0; Fig. 3B) (*P* = 0.0166; fold change = 0.74), sphingomyelin (d18:1/24:1, d18:2/24:0) (*P* = 0.0056; fold change = 0.74), sphingomyelin (d18:2/24:1, d18:1/24:2) (*P* = 0.0007; fold change = 0.71), and sphingosine 1‐phosphate (*P* = 0.022; fold change = 0.86; Fig. 3C). Increased levels of glycosphingolipids are associated with resistance to insulin signaling as manifested by reduced insulin‐stimulated glucose uptake. Two additional metabolites, glycosphingolipide: glycosyl‐N‐palmitoyl‐sphingosine (*P* = 0.088; fold change = 0.76), and glycosyl‐N‐stearoyl‐sphingosine (*P* = 0.0756; fold change = 0.60), were borderline statistically significant.

**Table 1:** Sphingolipid metabolism.

Metabolite	Fold change (MOTS‐c/Water)	*P* value	*q* value
Sphinganine	0.78	0.1213	0.3993
Sphinganine‐1‐phosphate**	**0.73**	**0.021**	**0.196**
N‐palmitoyl‐sphinganine (d18:0/16:0)	0.97	0.6427	0.6597
Palmitoyl dihydrosphingomyelin (d18:0/16:0)*	**0.86**	**0.0573**	**0.3038**
Behenoyl dihydrosphingomyelin (d18:0/22:0)	0.81	0.6022	0.6489
Palmitoyl sphingomyelin (d18:1/16:0)**	**0.77**	**0.0067**	**0.1694**
Stearoyl sphingomyelin (d18:1/18:0)**	**0.63**	**0.0043**	**0.1694**
Behenoyl sphingomyelin (d18:1/22:0)	0.85	0.7479	0.6902
Tricosanoyl sphingomyelin (d18:1/23:0)	0.93	0.7554	0.6902
Lignoceroyl sphingomyelin (d18:1/24:0)	1	0.9763	0.7374
Sphingomyelin (d18:1/14:0, d16:1/16:0)	0.8	0.2026	0.4862
Sphingomyelin (d18:2/14:0, d18:1/14:1)	0.62	0.1207	0.3993
Sphingomyelin (d18:1/15:0, d16:1/17:0)	0.9	0.4149	0.5865
Sphingomyelin (d18:2/16:0, d18:1/16:1)	0.83	0.1824	0.4641
Sphingomyelin (d18:1/17:0, d17:1/18:0, d19:1/16:0)	0.8	0.1202	0.3993
Sphingomyelin (d18:1/18:1, d18:2/18:0)**	**0.74**	**0.0166**	**0.1826**
Sphingomyelin (d18:1/20:0, d16:1/22:0)	0.66	0.2244	0.4896
Sphingomyelin (d18:1/20:1, d18:2/20:0)	0.72	0.1656	0.4492
Sphingomyelin (d18:1/21:0, d17:1/22:0, d16:1/23:0)	0.92	0.9414	0.7258
Sphingomyelin (d18:1/22:1, d18:2/22:0, d16:1/24:1)	0.76	0.3585	0.5592
Sphingomyelin (d18:2/23:0, d18:1/23:1, d17:1/24:1)	0.81	0.3329	0.5438
Sphingomyelin (d18:1/24:1, d18:2/24:0)**	**0.74**	**0.0056**	**0.1694**
Sphingomyelin (d18:2/24:1, d18:1/24:2)**	**0.71**	**0.0007**	**0.0727**
Sphingosine	0.95	0.8005	0.6929
Sphingosine 1‐phosphate**	**0.86**	**0.022**	**0.196**
N‐palmitoyl‐sphingosine (d18:1/16:0)	0.88	0.436	0.5946
N‐stearoyl‐sphingosine (d18:1/18:0)	0.92	0.7263	0.6829
Glycosyl‐N‐palmitoyl‐sphingosine*	**0.76**	**0.088**	**0.3449**
Glycosyl‐N‐stearoyl‐sphingosine*	**0.6**	**0.0756**	**0.3226**
Lactosyl‐N‐palmitoyl‐sphingosine	0.62	0.1816	0.4641

Bold indicates significant difference *P < 0.05 and **0.05 <*P* <0.10.

### Monoacylglycerol metabolism

**Figure 4:**
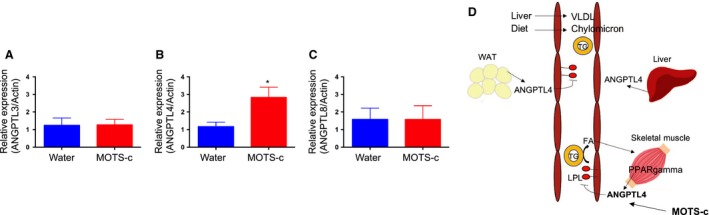
Fat accumulation in the muscle decreased suggesting reduced metabolic dysfunction. The mRNA expression levels of (A) ANGPTL3, (B) ANGPTL4, and (C) ANGPTL8 in water and MOTS‐c injected DIO mice muscle. (D) The pathways involved in monoacylglycerol biogenesis. Data are reported as mean ± SEM of six mice per group. ^*^ *P* < 0.05.

Monoacylglycerol, a byproduct of triacylglycerol breakdown, is hydrolyzed by membrane‐bound lipoprotein lipases (LPLs) for transport into the tissues. MOTS‐c–injected mice exhibited significantly lower monoacylglycerol metabolites when compared to water‐treated mice (Table 2). Metabolites significantly lowered by MOTS‐c included 2‐oleoylglycerol (18:1) (*P* = 0.0281; fold change = 0.68), 1‐linoleoylglycerol (18:2) (*P* = 0.0061; fold change = 0.74), 2‐linoleoylglycerol (18:2) (*P* = 0.0026; fold change = 0.65), 1‐linolenoylglycerol (18:3) (*P* = 0.0462; fold change = 0.59), and 1‐docosahexaenoylglycerol (22:6) (*P* = 0.0209; fold change = 0.49). Other metabolites in monoacylglycerol metabolism trended toward significance, such as 1‐oleoylglycerol (18:1) (*P* = 0.0757; fold change = 0.64) and 1‐palmitoleoylglycerol (16:1) (*P* = 0.1504; fold change = 0.67). MOTS‐c lowered the plasma monoacyglycerol levels, suggesting the possibility of reduced LPLs activity. The liver, muscle, and adipose tissues are major tissues expressing LPLs and that uptake fatty acid and monoacylglycerol for storage and fuel utilization (Zhang, 2016). LPLs activity is suppressed by ANGPTL3, 4, and 8, leading to the regulation of fatty acid and monoacylglycerol transport to the tissues (Zhang, 2016). We examined ANGPTL3, 4, and 8 mRNA expression in the liver, muscle, and adipose tissues. ANGPTL4 expression is lower in muscle, but not in other tissues (Figs. 4A–C and Fig. S2A–F). During high‐fat diet feeding, many chylomicrons occupy the bloodstream. Upon lipase activity, fatty acid and monoacylglycerol are transported into muscle and other tissues, and the accumulation of fats in the tissue cause dysfunction of the tissues. For example, in human and animal models, the increased lipid content of skeletal muscle is strongly associated with insulin resistance. The increased levels of ANGPTL4 presumably suppress the LPLs and prevent the accumulation of fats in the muscle in MOTS‐c–injected mice, leading to the improved insulin sensitivity (Fig. 4D). Indeed, MOTS‐c injected DIO mice showed improved insulin sensitivity (Lee et al., 2015).

**Table 2:** Monoacylglycerol metabolism.

Metabolite	Fold change (MOTS‐c/Water)	*P* value	*q* value
1‐Palmitoylglycerol (16:0)	0.62	0.5118	0.6199
2‐Palmitoylglycerol (16:0)	0.98	0.9234	0.7201
1‐Oleoylglycerol (18:1)*	**0.64**	**0.0757**	**0.3226**
2‐Oleoylglycerol (18:1)**	**0.68**	**0.0281**	**0.2246**
1‐Linoleoylglycerol (18:2)**	**0.74**	**0.0061**	**0.1694**
2‐Linoleoylglycerol (18:2)**	**0.65**	**0.0026**	**0.1424**
1‐Linolenoylglycerol (18:3)**	**0.59**	**0.0462**	**0.2779**
1‐Arachidonylglycerol (20:4)	0.87	0.2275	0.4896
1‐Docosahexaenoylglycerol (22:6)**	**0.49**	**0.0209**	**0.196**
1‐Dihomo‐linolenylglycerol (20:3)	0.71	0.1543	0.4363
1‐Palmitoleoylglycerol (16:1)*	**0.67**	**0.1504**	**0.4343**

Bold indicates significant difference *P < 0.05 and **0.05 < *P* < 0.10.

### Dicarboxylate metabolism

**Figure 5:**
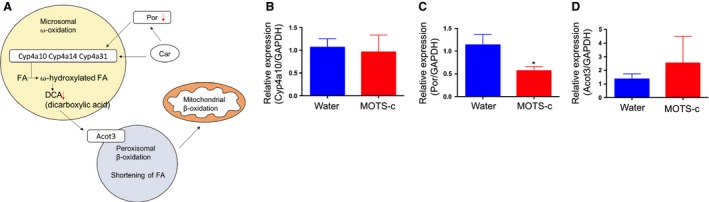
Omega oxidation is decreased in MOTS‐c–injected mice liver. (A) The dicarboxylic acid biosynthesis via omega oxidation. (B–D) The mRNA expression of the genes involved in the omega oxidation pathways in the liver. Data are reported as mean ± SEM of six mice per group. ^*^ *P* < 0.05.

MOTS‐c–injected mice showed significantly lower fatty acid dicarboxylates (DCAs; Table 3). Four metabolites in the fatty acid dicarboxylate pathway were statistically much lower in MOTS‐c–treated mice compared to water‐treated mice, including suberate (*P* = 0.0082; fold change = 0.6), sebacate (*P* = 0.0464; fold change = 0.58), undecanedioate (*P* = 0.0262; fold change = 0.58), and tetradecanedioate (*P* = 0.0445; fold change = 0.72). While not considered significant at *P* < 0.05, azelate (*P* = 0.0593; fold change = 0.63), dodecaneadioate (*P* = 0.0827; fold change = 0.75), and hexadecanedioate (*P* = 0.0769; fold change = 0.74) were all much lower in mice administered MOTS‐c. Dicarboxylic acids (DCAs) are formed from omega oxidation of monocarboxylic acids mainly in the liver and kidney (Wanders et al., 2011). Sebacate represents one of the more abundant dicarboxylates observed in plasma and tissues. When glucose metabolism is impaired, DCAs can be a fuel substrate immediately available for tissue energy requirements. Therefore, increases of dicarboxylates are generally thought to indicate dysfunction of normal mitochondrial and peroxisomal beta‐oxidation (Bergstrom et al., 1954; Y Yamakaya, 1950). Since the plasma dicarboxylates were lower in MOTS‐c‐treated mice, this suggests three possibilities: (1) greater efficiency in normal fatty acid oxidation, with a corresponding reduction in omega oxidation, (2) upregulation of utilization of DCAs in mitochondrial and peroxisomal oxidation, (3) increased uptake of DCAs to other tissues including skeletal muscles as an alternative fuel source. To address these possibilities, we checked the expression of three key omega oxidation‐related genes in the liver. Cytochrome P450 enzymes (eg. Cyp4a10, 14, 31) catalyze the fatty acid omega oxidation (Fig. 5A). Por, a rate‐limiting enzyme, is the electron donor for cytochrome P450 enzyme (Fig. 5A). ACOT3 is involved in the transport of DCA into peroxisomes and further transport out to mitochondria for beta‐oxidation (Fig. 5A). Cyp4a10, cytochrome P450 enzyme expression levels remained same (Fig. 5B). However, Por expression was reduced by 50% in the liver of MOTS‐c–injected mice (Fig. 5C). Deletion of the Por gene in a mouse model reduced hepatic P450 activity by more than 95%. Reduction of Por expression in MOTS‐c–injected mice probably affects p450 activity and reduces the production of DCAs. The DCAs transporter, Acot3, remained the same (Fig. 5D), suggesting the utilization of DCAs might remain the same in MOTS‐c–injected mice (null possibility 2). Taken together, the reduction of plasma DCAs in MOTS‐c–injected mice represents greater efficiency of normal beta‐oxidation. We previously showed that MOTS‐c increases beta‐oxidation in vitro and in vivo. Further studies whether DCAs’ uptake to other tissues including skeletal muscle increased during MOTS‐c administration will provide better understanding of the role of MOTS‐c in dicarboxylate metabolism.

**Table 3:** Fatty acid, dicarboxylate metabolism.

Metabolite	Fold change (MOTS‐c/Water)	*P* value	*q* value
2‐Hydroxyglutarate	1.06	0.8149	0.694
2‐Hydroxyadipate	1.35	0.3625	0.5611
Maleate	1	0.8116	0.694
Suberate (octanedioate)**	**0.6**	**0.0082**	**0.1694**
Azelate (nonanedioate)*	**0.63**	**0.0593**	**0.3096**
Sebacate (decanedioate)**	**0.58**	**0.0464**	**0.2779**
Undecanedioate**	**0.58**	**0.0262**	**0.2134**
Dodecanedioate*	**0.75**	**0.0827**	**0.3367**
Tetradecanedioate**	**0.72**	**0.0445**	**0.2779**
Hexadecanedioate*	**0.74**	**0.0769**	**0.3226**
Octadecanedioate	0.91	0.5903	0.6489
Eicosanodioate	0.8	0.1288	0.4039

Bold indicates significant difference *P < 0.05 and ^**^0.05 < *P* < 0.10.

## Discussion

**Figure 6:**
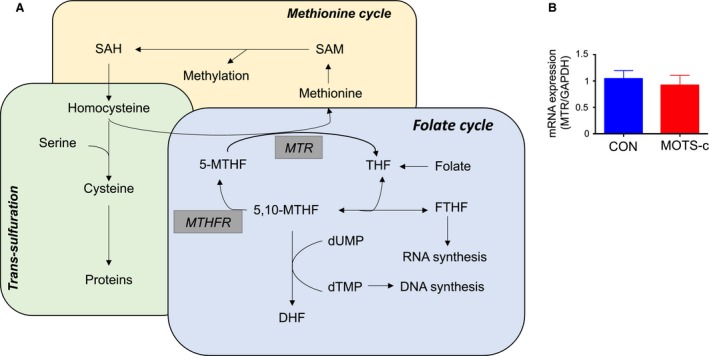
Folate cycle did not change in MOTS‐c injected DIO mice compared to control (A) The folate cycles and its related pathways. (B) The mRNA levels of MTR in the skeletal muscle.

Here, we performed plasma metabolite profiling from high‐fat diet‐induced obese mice injected with MOTS‐c for 3 days. MOTS‐c–injected mice showed lower glucose levels and insulin levels, suggesting MOTS‐c administration improves insulin sensitivity in high‐fat diet‐induced obese mice. Metabolite profiling showed that MOTS‐c decreases plasma metabolites in three independent pathways: sphingolipid metabolism, monoacylglycerol metabolism, and dicarboxylate metabolism. Interestingly, higher levels of these metabolites are associated with higher insulin resistance and metabolic syndromes. In addition to these three pathways, plasma oxidized glutathione was also lower in MOTS‐c injected group (Table S1), which may be consistent with reduced oxidative stress in cells and can promote cellular stress resistance against oxidative stress (Kim et al., 2018). We previously showed that the folate‐methionine cycle was reduced in MOTS‐c stably transfected HEK293 cells (Lee et al., 2015). The folate was reduced and 5‐methyltetrahydrofolate‐homocysteine methyltransferase (MTR) and methylenetetrahydrofolate reductase (MTHFR) mRNA expression went down (Lee et al., 2015). We would like to address whether MOTS‐c impacts the folate cycle in DIO mice. Folate was not detected in the plasma because the levels were below detection limit in DIO mice. The folate cycle, methionine cycle, and trans‐sulfuration cycle interact with each other (Fig. 6A). We, therefore, assessed the metabolites using the other two pathways. Methionine, S‐adenosylhomocysteine (SAH), and cysteine were not altered in MOTS‐c–treated mice (*data not shown*). We also examined MTR mRNA expression in the skeletal muscle and did not see a difference between MOTS‐c and water‐treated mice (Fig. 6B). Taken together, MOTS‐c does not appear to impact the folate cycle in DIO mice.

MOTS‐c is encoded from the 12S rRNA region of the mitochondrial genome and is a member of the mitochondrial‐derived peptide (MDP) family (Lee et al., 2015). Currently, eight MDPs have been proposed to be biologically active and to be protective or detrimental factors of certain conditions. We recently published the effect of other MDPs: humanin and SHLP2 on plasma metabolites in DIO mice (Mehta et al., 2019). Humanin and SHLP2 commonly regulate several metabolic pathways: glutathione, sphingolipid, gamma‐glutamyl cycle, and carnitine metabolism. Humanin and SHLP2 exert common biological functions (Mehta et al., 2019). Both peptides activate AKT, ERK, and STAT3 signaling pathways, and exhibit insulin sensitization as well as anti‐apoptosis effects (Cobb et al., 2016; Kim et al., 2016). On the other hand, MOTS‐c has both common and distinct metabolic regulation compared to these two MDPs. MOTS‐c uniquely modulates monoacylglycerol and dicarboxylate metabolism, although MOTS‐c decreases sphingolipid metabolism and glutathione metabolism similar to humanin and SHLP2. Similar to humanin and SHLP2, MOTS‐c modulates AKT, but it also modulates the AMPK pathway (Lee et al., 2015). The common signaling pathways among MDPs may be the reason for some of the similarity in metabolite changes that we have seen.

Sphingolipid metabolism was reduced in MOTS‐c–injected mice, but ceramide did not change much. However, S1P levels were lower in MOTS‐c–injected mice. S1P binds to the S1P receptor and inhibits insulin‐mediated AKT phosphorylation in the liver (Osawa et al., 2005). In muscle, S1P binds to S1P receptor and increase IL‐6 expression, which inhibits IRS activation upon insulin stimuli (Ross et al., 2013). Thereby, higher S1P induces insulin resistance. MOTS‐c–injected mice lower the plasma levels of S1P, resulting in the less binding to the S1P receptor, which in turn improve insulin sensitivity in DIO mice. S1P induces insulin resistance, but S1P also plays an important role in maintaining beta‐cell function. Further study whether or not the lower levels of S1P by MOTS‐c can impact beta‐cell function needs to be studied.

We interpreted that the reduction of monoacylglycerol metabolism is due to inhibition of LPLs by ANGPTL4 in skeletal muscle. It presumably prevents the fat accumulation in the skeletal muscle and improves insulin sensitivity. The levels of ANGPTLs in the liver and adipose tissues remained the same. However, the reduction of LPLs activity can cause the increase of triglyceride in the blood, although our metabolomics analysis panel does not include triglyceride and did not measure the levels of triglyceride in this plasma. The extra triglyceride from diet goes to the liver and is stored in the liver, which can cause fatty liver and hepatic steatosis. We previously showed that MOTS‐c injected DIO mice have dramatically reduced fatty liver (Lee et al., 2015). Although the triglyceride may enter more readily in the liver of MOTS‐c–injected mice due to the inhibited LPL activity, we speculate that MOTS‐c increases beta‐oxidation and overcomes the fat accumulation in the liver. Previously, we showed MOTS‐c increases beta‐oxidation in vitro. We also showed that the reduction of omega oxidation in the liver and reduction of DACs in the plasma from MOTS‐c–injected mice. This indicates the efficient beta‐oxidation occurs in the liver. Although we found that liver produce less DACs, we cannot rule out the possibility that the decrease in plasma DACs in the MOTS‐c injected mice is due to an increased uptake in other tissues such as the muscle. Healthy skeletal muscles have metabolic flexibility, so they can switch glucose and fat utilization depending on the context (Kelley, 2005). Nevertheless, the aged or stressed condition, skeletal muscles lose the metabolic flexibility and get fatigue soon during exercise (Kelley, 2005). DACs can be an alternative fuel source during the lack of metabolic flexibility (Salinari et al., 2006). We proposed that MOTS‐c is an exercise mimetic and may improve exercise capacity (Lee et al., 2016). MOTS‐c may increase the uptake of DACs in skeletal muscle during exercise in obese mice, reducing muscle fatigue and improving performance. It would be a great question in the future whether DACs uptake is increased in MOTS‐c–injected mice skeletal muscle and if MOTS‐c can improve the maximum exercise capacity in obese mice. DACs infusion has already been shown to improve exercise capacity and metabolism in obese and T2D patients (Salinari et al., 2006; Mingrone et al., 2013). MOTS‐c has now entered phase I clinical trials for hepatic steatosis. If MOTS‐c can increase the exercise capacity and improve metabolism in obese mice, MOTS‐c can be easily translated to the clinic.

## Conflict of Interest

Pinchas Cohen is a consultant and stockholder of CohBar Inc.

## Supporting information




**Figure S1**. Plasma metabolites differences between water and MOTS‐c injected DIO mice.

**Figure S2**. The ANGPTL 3,4, and 8 were not altered in the fat and the liver.

**Table S1**. Glutathionine metabolism
