# Supplementary figures and images for: The mitochondrial‐derived peptide MOTS‐c is a regulator of plasma metabolites and enhances insulin sensitivity
Source: Physiol Rep. 2019 Jul 10;7(13):e14171. doi: 10.14814/phy2.14171 (PMC6640593; doi:10.14814/phy2.14171)

A

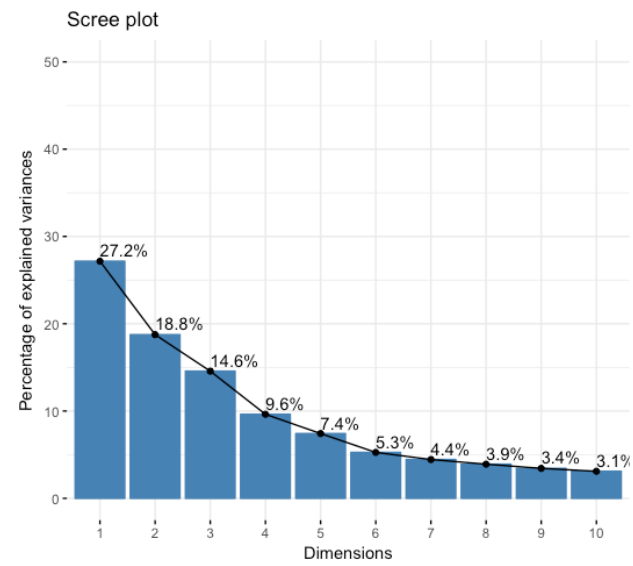

B

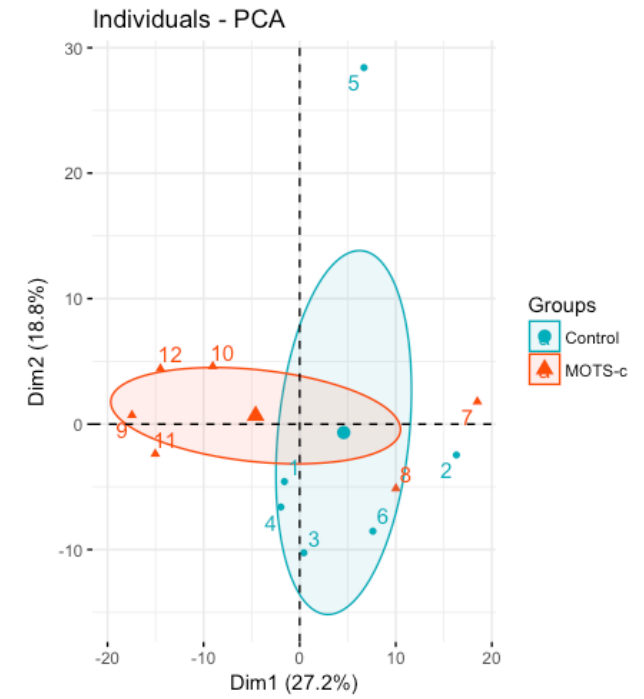

C

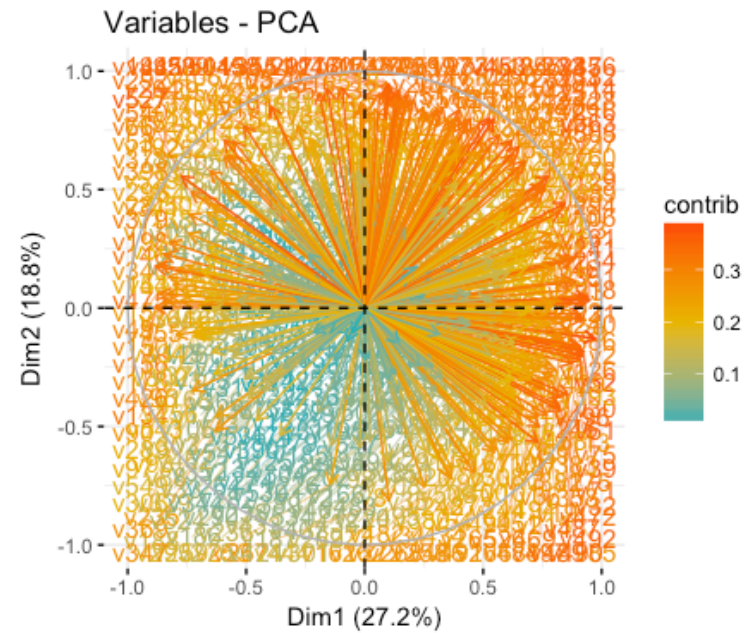

D

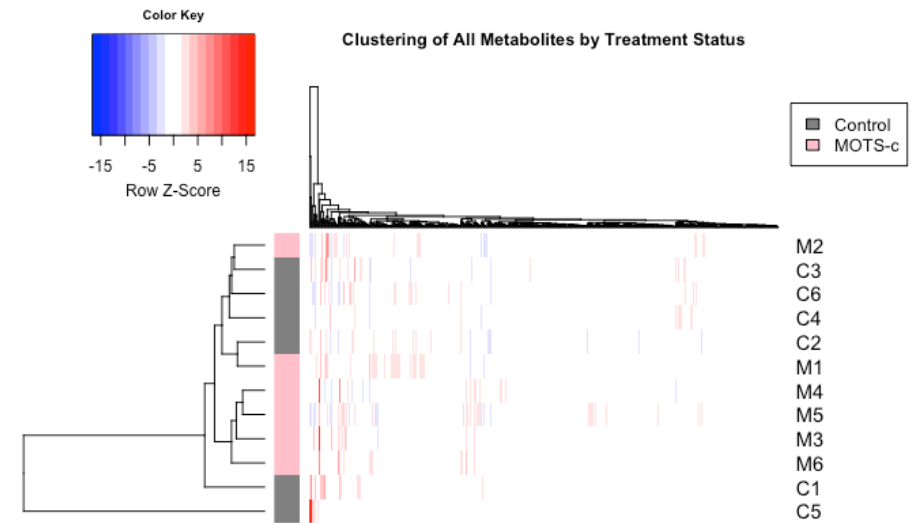

Supplement: Supplementary file 1 — Figure S1. Plasma metabolites differences between water and MOTS‐c injected DIO mice. [file PHY2-7-e14171-s001.pdf]

A

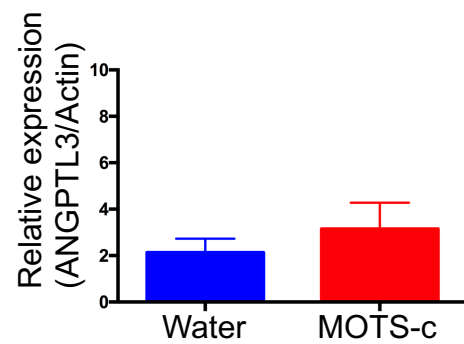

B

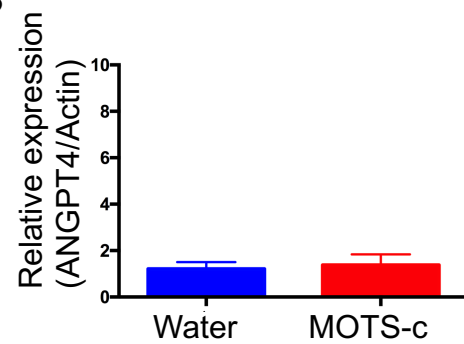

C

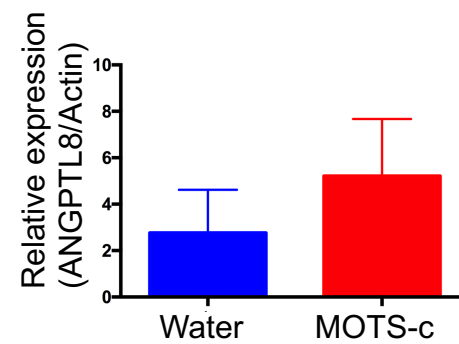

D

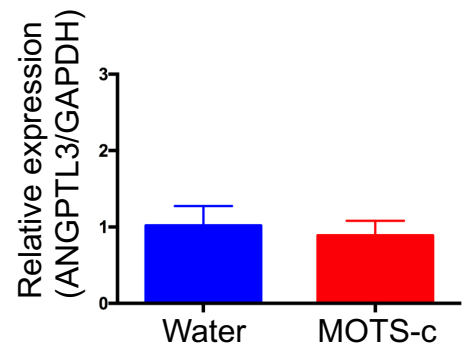

E

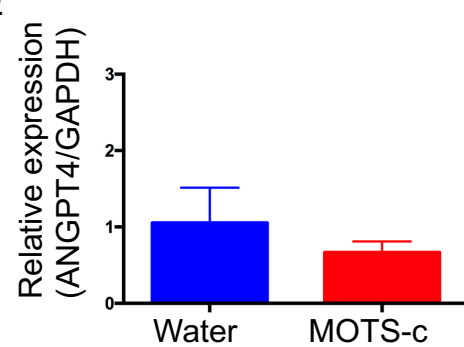

F

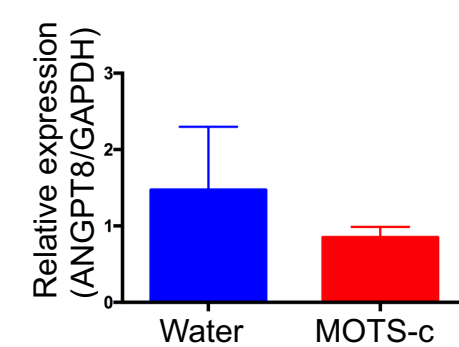

Supplement: Supplementary file 2 — Figure S2. The ANGPTL 3,4, and 8 were not altered in the fat and the liver. [file PHY2-7-e14171-s002.pdf]
