# Supplementary material for: The mitochondrial‐derived peptide MOTS‐c is a regulator of plasma metabolites and enhances insulin sensitivity
Source: Physiol Rep. 2019 Jul 10;7(13):e14171. doi: 10.14814/phy2.14171 (PMC6640593; doi:10.14814/phy2.14171)
Supplement: Supplementary file 3 — Table S1. Glutathionine metabolism [file PHY2-7-e14171-s003.docx]

| Supplemental table 1. Glutathionine Metabolism | | | |
| --- | --- | --- | --- |
| Metabolite | Fold Change (MOTS-c/Water) | p value | q value |
| **glutathione, oxidized (GSSG) *** | **0.58** | **0.0066** | **0.1694** |
| cysteine-glutathione disulfide | 0.89 | 0.4309 | 0.5946 |
| **S-methylglutathione *** | **0.42** | **0.0006** | **0.0727** |
| **5-oxoproline *** | **0.83** | **0.0083** | **0.1694** |
| **ophthalmate *** | **0.38** | **0.0012** | **0.0951** |
| * p < 0.05 and ** 0.05<p<0.10 |  |  |  |
